# Enhancing the Separation Performance of Cellulose Membranes Fabricated from 1-Ethyl-3-methylimidazolium Acetate by Introducing Acetone as a Co-Solvent

**DOI:** 10.3390/membranes14090202

**Published:** 2024-09-23

**Authors:** Luying Chen, Dooli Kim, Wiebe M. de Vos

**Affiliations:** 1 Membrane Science and Technology, MESA+ Institute for Nanotechnology, University of Twente, P.O. Box 217, 7500 AE Enschede, The Netherlands; lychen@szjm.edu.cn; 2 Key Lab of Science and Technology of Eco-Textile, Ministry of Education, College of Chemistry, Chemical Engineering and Biotechnology, Donghua University, Shanghai 201620, China; 3 Sustainable Process Technology, Faculty of Science and Technology, University of Twente, P.O. Box 217, 7500 AE Enschede, The Netherlands

**Keywords:** cellulose, membranes, co-solvent introduction, water filtration, fouling behavior

## Abstract

Cellulose, a sustainable raw material, holds great promise as an ideal candidate for membrane materials. In this work, we focused on establishing a low-cost route for producing cellulose microfiltration membranes by adopting a co-solvent system comprising the ionic liquid 1-ethyl-3-methylimidazolium acetate ([EMIM]OAc) and acetone. The introduction of acetone as a co-solvent into the casting solution allowed control over the viscosity, thereby significantly enhancing the morphologies and filtration performances of the resulting cellulose membranes. Indeed, applying this co-solvent allowed the water permeability to be significantly increased, while maintaining high rejections. Furthermore, the prepared cellulose membrane demonstrated excellent fouling resistance behavior and flux recovery behavior during a challenging oil-in-water emulsion filtration. These results highlight a promising approach to fabricate high-performance cellulose membranes.

## 1. Introduction

Cellulose is an abundant and renewable biopolymer derived from nature. It has characteristics of biodegradability, low cost, high hydrophilicity and non-toxicity, and is highly suitable for various potential applications, especially in the preparation of separation membranes [1,2,3,4,5]. However, due to the structure of cellulose, which has stable glycosidic bonds and abundant intermolecular hydrogen bonds, it is insoluble in water and most organic solvents [6,7,8,9,10]. Numerous solvent systems have been studied to dissolve and process cellulose such as N,N-dimethylacetamide/lithium chloride (DMAc/LiCl) [11,12], dimethyl sulfoxide/tetrabutylammonium fluoride (DMSO/TBAF) [13], LiSCN·2H_2_O [1], N-methyl morpholineN-oxide (NMMO) [14,15], and NaOH/urea/H_2_O [16]. However, these solvent systems come with drawbacks, such as volatility, toxicity, instability, and flammability [17]. Consequently, there is still a need to utilize solvents with lower toxicity, excellent recyclability, and reusability for the dissolution and processing of cellulose. 

Ionic liquids (ILs) are composed of organic cations and inorganic/organic anions and are defined as salts which have a melting temperature below 100 °C [18,19,20]. Due to their unique chemical structure, they usually have a strong hydrogen bonding and coulombic intermolecular force. ILs are widely acclaimed for their environmentally friendly properties and unique features. ILs do not produce harmful volatile organic compounds (VOCs) and have negligible vapor pressure, good thermal stability, chemical stability, recyclability, and non-flammability and therefore often referred to as green solvents [21,22,23]. 

ILs have been widely used for dissolving cellulose [24,25,26] and show good solubility, stability and recyclability for cellulose, making positive contributions to sustainable industry and environmental protection [27]. The dissolution behavior of cellulose varies in different ILs. For example, Kosan et al. [28] reported that ILs containing acetate anions demonstrate higher efficiency in the cellulose dissolution process compared to those with chloride anions. Common types of ILs include 1-butyl-3-methylimidazolium chloride [BMIM]Cl [29], 1-ethyl-3-methylimidazolium acetate [EMIM]OAc [30], and 1-allyl-3-methylimidazolium chloride [AMIM]Cl [31]. Among them, [EMIM]OAc is regarded as an excellent candidate for directly dissolving cellulose due to the high alkalinity of the anion and the high acidity of the cation. Nevstrueva et al. [32] used [EMIM]OAc as a solvent and deionized water as a nonsolvent to prepare cellulose filtration membranes via phase-inversion. The membranes fabricated by changing the coagulation bath temperature showed different morphologies and filtration properties. Nevertheless, the cellulose solution obtained by dissolving cellulose in [EMIM]OAc exhibits a relatively high viscosity, which is not beneficial for the subsequent processing step, namely the fabrication of cellulose membranes. Using a co-solvent system could not only efficiently reduces the viscosity of cellulose/IL solution but also introduces additional tuning parameter to control the morphology and filtration capabilities of the membranes.

Herein, we prepared cellulose membranes from ionic liquid solution with acetone as co-solvent. Acetone was introduced to reduce the viscosity of the cellulose/[EMIM]OAc solution, thereby simplifying both solution preparation and membrane manufacturing. Acetone is also a nonsolvent for cellulose, which can also be used as a nonsolvent bath to prepare cellulose membranes by nonsolvent induced phase separation (NIPS) [33]. Thus, it is important to ensure the stability of the cellulose/[EMIM]OAc/acetone mixture by controlling the added amount of acetone within a suitable range. The effect of acetone addition on the viscosity of cellulose/[EMIM]OAc/acetone mixture was quantitatively tested. The influence of the acetone addition on the morphology of the created cellulose membranes were investigated by scanning electron microscopy (SEM) technique. Furthermore, a number of oil-in-water filtration experiments were performed to investigate the effect of the acetone amount on the separation properties and fouling behavior of the membranes. The experimental results showed that the co-solvent system can affect the phase inversion during the membrane formation, thereby controlling the membrane structure and separation performance. This work provides a simple and efficient method for fabrication of cellulose membranes, ideal for separating oil-in-water emulsions, from cheap materials. The resulting cellulose membranes, demonstrating high porous structure, water permeability, and filtration efficiency, have great potential to be applied in water treatment.

## 2. Experimental Section

### 2.1. Materials

Avicel PH-101 microcrystalline cellulose, n-hexadecane, sodium dodecyl sulfate, acetone, and Oil Red EGN dye were purchased from Sigma-Aldrich, Zwijndrecht, The Netherlands. 1-Ethyl-3-methylimidazolium acetate ([EMIM]OAc) with purity equal to or higher than 95% was supplied by Sigma-Aldrich, Zwijndrecht, the Netherlands. All water used was Milli-Q water with specific resistivity 18.2 MΩ cm at 26.1 °C.

### 2.2. Preparation of the Casting Solution

Initially, the cellulose solutions were prepared by directly dissolving cellulose in [EMIM]OAc at 90 °C for 12 h. Subsequently, acetone was introduced to the cellulose solutions and the applied temperature was reduced to 50 °C to avoid the loss of acetone. In total four solutions were fabricated to investigate the effect of acetone on the membrane performance, with mass ratios of [EMIM]OAc to acetone set at 1:0, 4:1, 3:1, and 2:1, while maintaining an overall cellulose concentration of 10 wt%. The solutions were then stirred until they became homogeneous. Table 1 shows the compositions of all solutions. 

### 2.3. Membrane Fabrication

**Figure 1:**
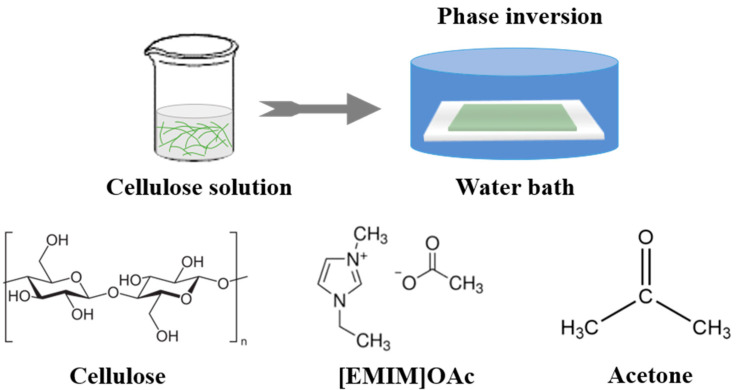
Fabrication process of cellulose membranes.

The cellulose flat membranes were formed at room temperature using non-solvent induced phase separation method as shown in Figure 1. The cellulose solutions were cast onto a non-woven fabric on a glass plate with a casting knife thickness of 400 µm and promptly immersed in the deionized water as a non-solvent to induce phase separation. Finally, the membranes were thoroughly washed with deionized water, and stored in deionized water before conducting any characterization and testing procedure. To prevent pore collapse during the drying process, membranes were firstly soaked in a 50 wt% glycerol/50 wt% water solution overnight and after air-drying in the fumehood, the prepared membranes were stored at room temperature until further study. Notably, the membranes were washed for several hours before the filtration experiments to completely remove glycerol, preventing any effect on the filtration performance of the membranes.

### 2.4. Characterization

#### 2.4.1. Viscosity Measurements

The viscosity of the cellulose solutions was measured using a HAAKE Viscotester 550 Rotational Viscometer (ThermoFisher Scientific, Waltham, USA). Around 15 mL of the solution was carefully poured into a standardized cylinder (SV-DIN) and subsequently a spindle was affixed to the viscometer. The dynamic viscosity of the solution was then measured at a temperature of 25 °C, with shear rates ranging from 1.1 to 258 s^−1^.

#### 2.4.2. Characterization of Membrane Structure

In order to observe the morphology of the membranes, the membranes were prepared by directly casting cellulose solution on the glass plate. The surface and cross-sectional morphologies of the membranes were analyzed using scanning electron microscopy (JSM6010A, JEOL, Tokyo, Japan). For cross-sectional images, the membranes were fractured using liquid nitrogen to preserve the pore structure collapse and then mounted on the sample holders. After dried under vacuum at 30 °C for 24 h, the samples were sputtered with 5 nm Pt/Pd coating (Quorum Q150T ES) before capturing SEM images. 

#### 2.4.3. Pure Water Permeability Test and Pore Size Measurements

Cellulose membranes with a diameter of 25 mm and circular disk shape were prepared and placed in a dead-end filtration setup to study the water transport ability. The applied pressure was 2 bar and the weight of the collected permeate was monitored over time to determine the pure water permeability. All water permeability tests were carried out in triplicate (*n* ≥ 3) as a minimum, and for each measurement, fresh membranes were applied. The pure water permeability was calculated using Equation (1):

P (L·m^−2^·h^−1^ bar^−1^) = J_w_/Δp (1)

where J_w_ represents the water flux (L·m^−2^·h^−1^) and Δp is the transmembrane pressure (bar).

The membrane pore size was characterized using a Porolux 500 capillary flow porometer (IB-FTGmbH, Berlin, Germany). Specifically, the membranes (typical size 25 mm in diameter) were firstly impregnated in perfluorotributylamine (Fluorinert FC-43, 3M Belgium) for 10 min. Then, the membranes were removed from the liquid, placed in the porometer, and measured under N_2_ atmosphere.

#### 2.4.4. Oil-in-Water Emulsion Retention Test

For the evaluation of retention performance with oil-in-water emulsion using a dead-end filtration setup, the emulsion was prepared according to a specific protocol [34]. In summary, n-hexadecane (100 mg/L) was blended with Oil Red EGN dye (20 mg/L) as a clear marker for the emulsion droplets and the blended solution was gradually introduced into an SDS solution (463 mg/L), while stirring continuously at 14,000 rpm for 20 min. Following this process, an emulsion featuring oil droplets in the size range of 3–4 µm was successfully produced. The separation test was carried out under a transmembrane pressure of 2 bar. The permeate solutions were collected and analyzed using a UV–vis spectrophotometer at λ_max_ = 521 nm (PerkinElmer Lambda 850), representing the maximum absorbance wavelength of Oil Red EGN. A minimum of three samples were tested, and the retention R (%) was calculated using the following Equation (2):

R(%) = (C_f_ − C_p_)/C_f_ × 100 (2)

Here, C_f_ and C_p_ represent the concentration of oil droplets in the feed and permeate solutions, respectively.

#### 2.4.5. Fouling during Oil-in-Water Emulsion Filtration Test

In order to evaluate the fouling resistance behavior of cellulose membranes, the following protocol was applied for all membranes and their performances were compared. The pure water permeability was measured first. Secondly, the prepared oil-in-water emulsion was filled in, and the emulsion permeability was measured. To see the fouling effect, the pure water permeability was measured again and the fouling ratio was calculated by comparing pure water permeability before and after applying oil-in-water emulsion filtration. Finally, after gentle washing of the membrane surface with pure water, pure water permeability was retested to evaluate the flux recovery.

## 3. Results and Discussion 

### 3.1. Characterization of the Casting Solutions

In the process of preparing the membranes, the cellulose polymer concentration is crucial in determining the mechanical strength of the resultant cellulose membrane. For this reason, a higher polymer concentration of 10 wt% cellulose in all polymer solutions was maintained, while to observe the co-solvent effect, 10 wt% cellulose was dissolved in either pure [EMIM]OAc or [EMIM]OAc/acetone mixtures. An optimal casting solution should possess a suitable viscosity. By using the co-solvent system consisting of [EMIM]OAc and acetone, the dynamic viscosity of the cellulose solution can be effectively decreased. Notably, the polymer solution at an [EMIM]OAc and acetone ratio of 1:0 (pure [EMIM]OAc) was homogeneous, and polymer solutions with ratios of 4:1 and 3:1 were also homogeneous. However, the solution with the ratio of 2:1 exhibited non-homogeneous behavior, and we observed that if the amount of the co-solvent acetone exceeded the cellulose solubility, the solubility of cellulose was significantly reduced. The true solvent for cellulose dissolution is [EMIM]OAc and, as a co-solvent, acetone may weaken the interaction between cellulose and [EMIM]OAc. However, if the ratio of [EMIM]OAc to acetone is maintained below the threshold, which would be between 3:1 and 2:1, the solvent mixture can dissolve cellulose completely and even increase the speed of cellulose dissolution by reducing the mass transfer limit. For further studies, cellulose membranes with the [EMIM]OAc and acetone ratios of 1:0, 4:1, 3:1 were made. 

**Figure 2:**
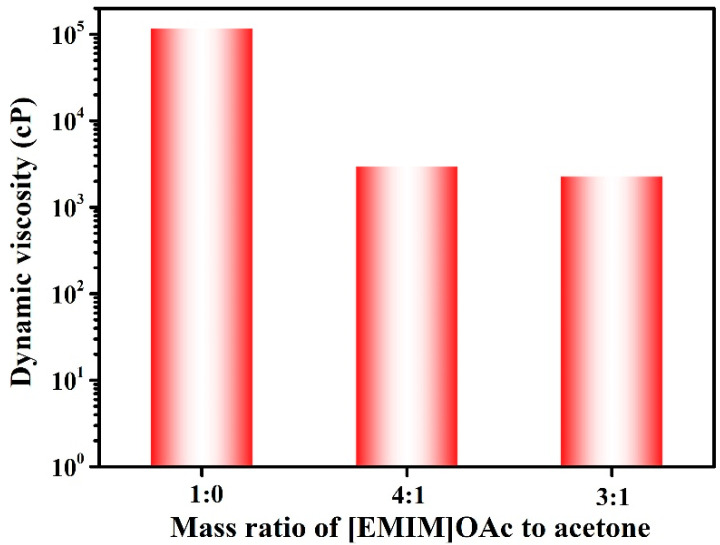
Dynamic viscosity of the cellulose/[EMIM]OAc/acetone casting solution solutions at various mass loading of [EMIM]OAc to acetone.

To prepare cellulose solutions for membrane fabrication, their viscosities should be maintained within a processible range. As shown in Figure 2, the viscosity of the cellulose/[EMIM]OAc solution was very high (118,000 cP), primarily attributed to the coordination of the cellulose hydroxyl groups with [EMIM]^+^ and [OAc]^−^ ions [35]. As acetone reduced the monomeric friction coefficient (the friction experienced by individual polymer chains as they move in a solution) in cellulose/[EMIM]OAc solutions and did not change the conformation of cellulose chains in the solvent, the viscosities of cellulose/[EMIM]OAc/acetone solutions decreased dramatically (Figure 2) [36]. On the one hand, the incorporation of acetone not only enhanced the processability of cellulose/[EMIM]OAc solutions, but also reduced the processing cost since acetone has lower cost than [EMIM]OAc. On the other hand, the reduction in casting solution viscosity increased the rate of solvent and non-solvent exchange, which greatly affects the precipitation kinetics, leading to the formation of membranes with different pore structures. 

### 3.2. Characterization of Fabricated Membranes 

**Figure 3:**
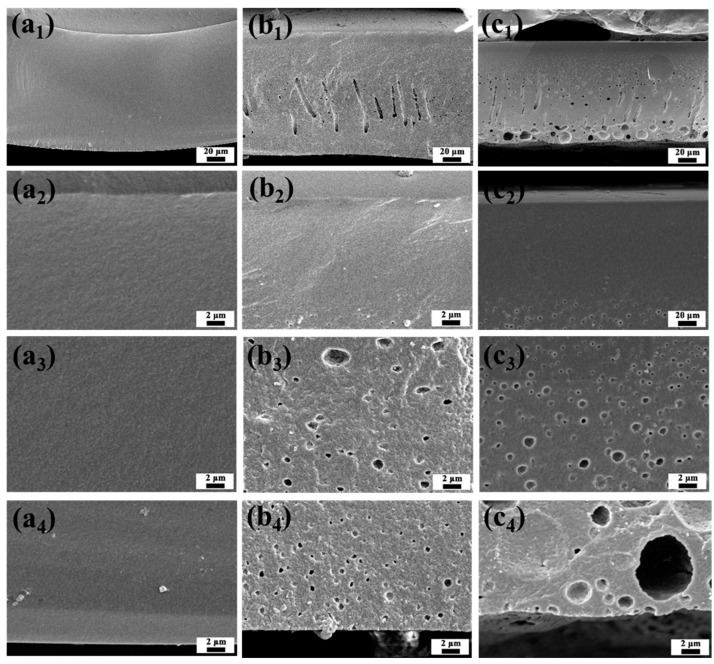
Cross-sectional SEM images of membranes showing the effect of the acetone loading on membrane porous structure: (**a_1_**–**a_4_**) IL:acetone = 1:0; (**b_1_**–**b_4_**) IL:acetone = 4:1; (**c_1_**–**c_4_**) IL:acetone = 3:1. (**a_1_**,**b_1_**,**c_1_**) show the overall cross-section images; (**a_2_**,**b_2_**,**c_2_**) are enlarged view of the top part; (**a_3_**,**b_3_**,**c_3_**) are enlarged view of the middle part; and (**a_4_**,**b_4_**,**c_4_**) are enlarged view of the bottom part.

**Figure 4:**
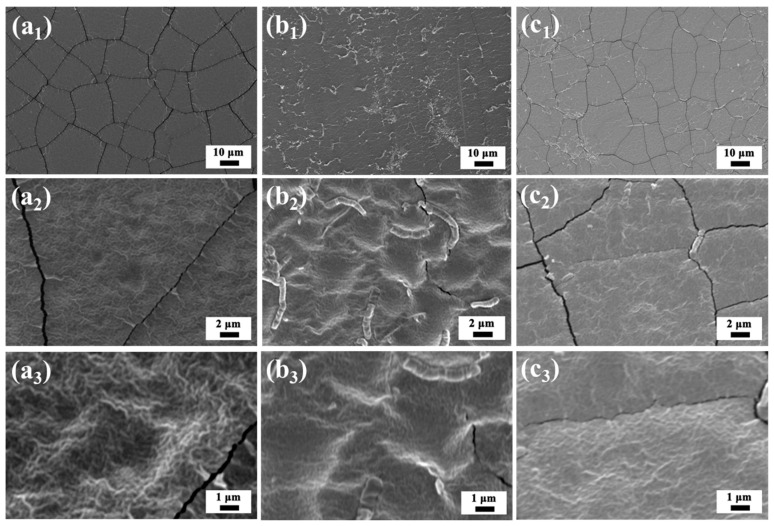
Top surface SEM images of membranes showing the effect of the acetone loading on membrane porous structure: (**a_1_**–**a_3_**) IL:acetone = 1:0; (**b_1_**–**b_3_**) IL:acetone = 4:1; and (**c_1_**–**c_3_**) IL:acetone = 3:1.

It was observed that the cellulose membranes prepared using the cellulose/[EMIM]OAc casting solution required several minutes to complete the precipitation. The high viscosity of cellulose/[EMIM]OAc solution led to a very dense overall structure of cellulose membranes, as shown in Figure 3a_1_, exhibiting no visible pores on the surface or cross-section microstructure (Figure 3a_1_–a_4_ and Figure 4a_1_–a_3_). In contrast, the cellulose membranes fabricated from the cellulose/[EMIM]OAc/acetone casting solution exhibited instant precipitation, promoting the formation of different pore structures, comprising a relatively dense top layer and a porous cross-section microstructure (Figure 3b,c). The appearance of the membrane also gradually changed from transparent to translucent upon introducing acetone as the co-solvent system due to the difference in the pore structure. Moreover, as the amount of acetone increased, larger and a greater number of pores were formed as observed in the cross-sectional structure of the membrane. This indicates that the incorporation of acetone is a critical parameter for controlling membrane structure. In addition, the top surface of all the membranes was free of defects as shown in Figure 4b,c, indicating that the membranes are suitable for water treatment, as the presence of defects would significantly reduce the retention capacity.

### 3.3. Filtration Performance of the Cellulose Membrane

**Figure 5:**
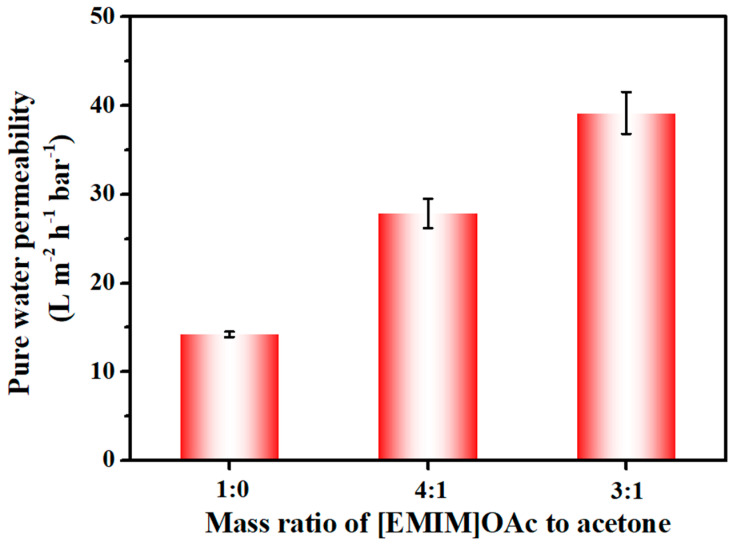
Pure water permeability of the membranes prepared in different acetone loadings in the casting solution.

The filtration performance of cellulose membranes was evaluated in terms of pure water permeability and retention properties of oil-in-water emulsions. The pure water permeability of the membranes is a key parameter for comprehending both membrane porosity and hydrophilicity. Figure 5 showed the pure water permeability of the cellulose membranes concerning the acetone loading in the casting solution. As mentioned earlier, the resulting cellulose membrane with pure [EMIM]OAc showed almost no pore morphology in the cross-section part, resulting in a low water permeability of approximately 14 L m^−2^ h^−1^ bar^−1^. The addition of acetone resulted in membranes with a notably more porous structure, thereby leading to improved water permeability values. The pure water permeability was approximately 28 L m^−2^ h^−1^ bar^−1^ at a solvent mixture ratio of 4:1 ([EMIM]OAc: acetone) and approximately 39 L m^−2^h^−1^bar^−1^ at a mass ratio of 3:1. The nearly 178% increase in water permeability was attributed to the porous pore structure induced by the co-solvent system, which is beneficial for the practical water treatment procedure. The biggest pore size (bubble point in porometer) of the cellulose membranes prepared by pure [EMIM]OAc and the co-solvent system were 0.068 μm and 0.48 μm, respectively, indicating that the pores of cellulose membranes prepared using the co-solvent were both larger and within the range of microfiltration.

**Figure 6:**
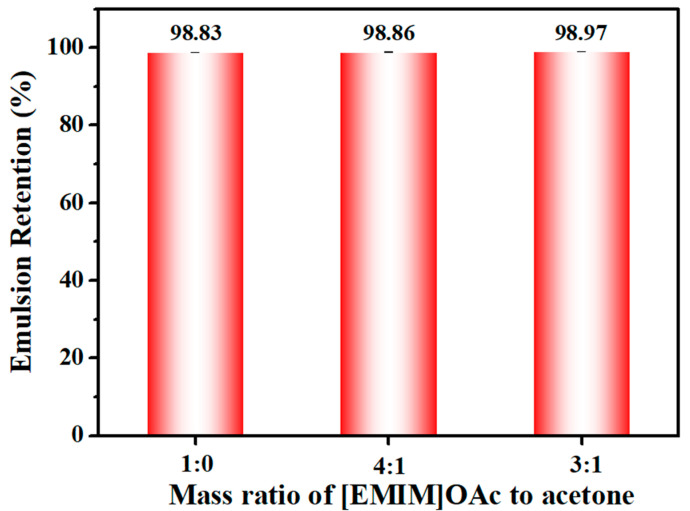
Emulsion retention of the membranes prepared in different acetone loadings in the casting solution.

The filtration efficiency of the cellulose membranes was evaluated by conducting oil-in-water emulsion retention tests in a dead-end cell configuration. The oil droplet (3–4 µm) retention was higher than 98 wt% regardless of whether the membranes were prepared from casting solutions contained acetone (Figure 6). The high retention was attributed to the dense top surface of the cellulose membranes, effectively retaining oil droplets while allowing only water to permeate through. Such high retentions also indicate that these membranes exhibit good mechanical stability, and have no defects on the membrane surface. To summarize, the cellulose membranes with high permeability that maintain higher emulsion selectivity can be obtained by regulating the phase separation process using a co-solvent system.

### 3.4. Fouling Behavior Study

**Figure 7:**
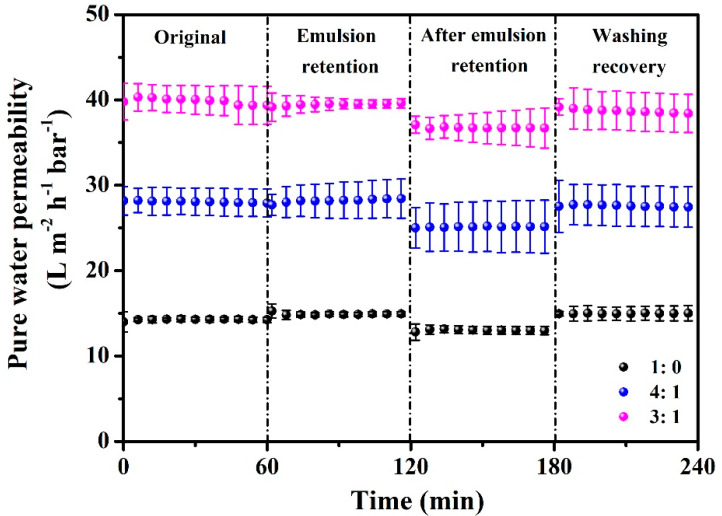
Original pure water permeability, permeability during the emulsion retention process, recovered water permeability after emulsion retention, and recovered water permeability after being washed with water for the membranes prepared in different acetone loadings in the casting solution.

During the emulsion separation process, the entrapment of oil droplets occurred, leading to membrane contamination and subsequently reducing water permeability. As shown in Figure 7 and Table 2, the decline in membrane permeability after emulsion retention tests was caused by oil contamination. Fortunately, the permeability of these membranes could be fully recovered after simple washing with pure water. These results demonstrate that all membranes have anti-fouling behavior and show the applicability in oil-in-water emulsion separation.

## 4. Conclusions

In this study, we prepared cellulose membranes with non-solvent induced phase inversion. Phase inversion kinetics can be controlled by changing the composition of the casting solution using a co-solvent system. The results show that increasing the acetone solvent loading in the casting solution led to faster phase inversion kinetics, and the microfiltration cellulose membranes with dense top layers and porous support layers can be obtained. The optimum casting solution was one with a mass ratio of acetone to [EMIM]OAc of 3:1. The prepared cellulose membranes possessed high water permeability, with a 178% increase compared to the cellulose membranes prepared from pure [EMIM]OAc. Additionally, the cellulose membranes showed emulsion droplet retention greater than 98% and could be used to effectively treat oily water streams. This work demonstrates that by applying co-solvent, the prepared cellulose membranes can be mechanically stable and highly water-permeable with high oil rejection capabilities, and thus can potentially be used in water treatment applications.

## Figures and Tables

**Table 1:** The composition of polymer solution prepared in this study.

	Cellulose	[EMIM]OAc	Acetone
1:0	10 wt%	90 wt%	0 wt%
4:1	10 wt%	72 wt%	18 wt%
3:1	10 wt%	67.5 wt%	22.5 wt%
2:1	10 wt%	60 wt%	30 wt%

**Table 2:** Permeability of membranes prepared in this study.

Membrane	Initial Pure Water Permeability (L m^−2^ h^−1^ bar^−1^)	Fouling Rate (%)	Flux Recovery (%)
1:0	14.30	91.12	87.03
4:1	28.09	89.75	91.44
3:1	40.04	91.68	95.01

## Data Availability

Data will be made available upon request to the corresponding authors.
